# Explaining high and low performers in complex intervention trials: a new model based on diffusion of innovations theory

**DOI:** 10.1186/s13063-015-0755-5

**Published:** 2015-05-31

**Authors:** Heather McMullen, Chris Griffiths, Werner Leber, Trisha Greenhalgh

**Affiliations:** Centre for Primary Care and Public Health, Queen Mary University of London, 58 Turner Street, London, E1 2AB UK; Department of Primary Care Health Sciences, University of Oxford, New Radcliffe House, Radcliffe Observatory Quarter, Woodstock Road, Oxford, OX2 6GG UK

**Keywords:** Complex interventions, Diffusion of innovations, Point of care testing

## Abstract

**Background:**

Complex intervention trials may require health care organisations to implement new service models. In a recent cluster randomised controlled trial, some participating organisations achieved high recruitment, whereas others found it difficult to assimilate the intervention and were low recruiters. We sought to explain this variation and develop a model to inform organisational participation in future complex intervention trials.

**Methods:**

The trial included 40 general practices in a London borough with high HIV prevalence. The intervention was offering a rapid HIV test as part of the New Patient Health Check. The primary outcome was mean CD4 cell count at diagnosis. The process evaluation consisted of several hundred hours of ethnographic observation, 21 semi-structured interviews and analysis of routine documents (e.g., patient leaflets, clinical protocols) and trial documents (e.g., inclusion criteria, recruitment statistics). Qualitative data were analysed thematically using—and, where necessary, extending—Greenhalgh et al.’s model of diffusion of innovations. Narrative synthesis was used to prepare case studies of four practices representing maximum variety in clinicians’ interest in HIV (assessed by level of serological testing prior to the trial) and performance in the trial (high vs. low recruiters).

**Results:**

High-recruiting practices were, in general though not invariably, also innovative practices. They were characterised by strong leadership, good managerial relations, readiness for change, a culture of staff training and available staff time (‘slack resources’). Their front-line staff believed that patients might benefit from the rapid HIV test (‘relative advantage’), were emotionally comfortable administering it (‘compatibility’), skilled in performing it (‘task issues’) and made creative adaptations to embed the test in local working practices (‘reinvention’). Early experience of a positive HIV test (‘observability’) appeared to reinforce staff commitment to recruiting more participants. Low-performing practices typically had less good managerial relations, significant resource constraints, staff discomfort with the test and no positive results early in the trial.

**Conclusions:**

An adaptation of the diffusion of innovations model was an effective analytical tool for retrospectively explaining high and low-performing practices in a complex intervention research trial. Whether the model will work prospectively to predict performance (and hence shape the design of future trials) is unknown.

**Trial registration:**

ISRCTN Registry number: ISRCTN63473710. Date assigned: 22 April 2010.

## Background

### Introduction

A *complex intervention* is defined by the Medical Research Council (MRC) as comprising multiple elements, all of which seem essential but whose ‘active ingredient’ may be difficult to specify; they typically operate at multiple levels (individual, team, organisation) [1–3]. Such interventions include new tests and treatments that create opportunities for changing how services are delivered (e.g., near-patient testing that potentially allows diagnoses to be made in primary care that were previously possible only in secondary care).

Much health services research consists of developing complex interventions and testing them in randomised controlled trials (RCTs). The MRC framework proposes five phases (0 to 4), including developmental and pilot work, the trial itself and an evaluation of post-trial implementation in the ‘real world’ [4]. Complex intervention trials generally require a cluster design (in which the organisation or service team is the unit of randomisation) and are studied through a pragmatic lens (i.e., seeking to replicate usual care as delivered by the staff and through systems in participating organisations) rather than an explanatory one (i.e., seeking to produce abstracted theoretical models of efficacy with an emphasis on scientific purity) [5, 6].

A growing theoretical and methodological literature addresses the question whether interventions that are *complex* can legitimately be tested using experimental designs in which they are conceptualised as a clearly defined set of inputs implemented in a controlled way with attention to mediating and moderating variables [4, 7, 8] or whether their complexity requires a more ecological conceptualisation as ‘events in systems’ and (therefore) developmental rather than experimental research designs [9–12]. Either way, a key focus of study is the interaction between the complex intervention and the local settings in which it is implemented [3, 13, 14].

An important concept is the idea of a theoretical ‘hard core’ of a complex intervention (elements that cannot be compromised without invalidating the trial) and a flexible ‘soft periphery’ (elements of the intervention that can and should be adapted locally to optimise acceptance and embedding) [15, 16]. In any complex intervention trial, each unit (e.g., participating organisation or team) will implement the intervention differently, so a component of trial quality is ensuring fidelity of the theoretical core [2, 15].

The emerging science of process evaluation uses qualitative research alongside a RCT to capture the experiences of staff and patients, illuminate tasks and processes, explore model–reality gaps and develop and test theory [17, 18]. Such approaches can be used both retrospectively (to explain successes and failures) and prospectively (to inform further refinement of the intervention). Specific theoretical lenses applied in this context include normalisation process theory [19] and realist evaluation [20], though the latter has been contested [21].

One approach that has not previously been used to study the process of implementing a complex intervention in a RCT is diffusion of innovations theory. Originally developed by Everett Rogers in the 1950s to explain the adoption and spread of innovations by individuals in a social network [22], the theory was later extended by Greenhalgh et al. to address the assimilation and implementation of service-level innovations in health care organisations [23]. Greenhalgh et al*.*’s definition of an innovation as “a novel set of behaviors, routines, and ways of working that are directed at improving health outcomes, administrative efficiency, cost effectiveness, or users’ experience and that are implemented by planned and coordinated actions” ([23], p. 582) agrees strongly with the MRC definition of a complex intervention (paragraph 1). It follows that the multi-level model developed by Greenhalgh et al. to study the adoption (and non-adoption and abandonment) of innovations may also prove useful for explaining variation in implementation success in complex intervention trials.

In this article, we apply Greenhalgh et al.’s model to a retrospective process evaluation of a complex intervention to introduce rapid HIV testing in a general practice setting. Below we summarise the trial and introduce the diffusion of innovations model and then describe our methodology, findings and conclusions. In the Discussion section, we offer preliminary suggestions for using the diffusion of innovations model prospectively to optimise organisational participation in trials.

### The trial of rapid HIV testing in general practice

A summary of the rationale, methodology and findings of this trial have been published elsewhere [24, 25]. Briefly, general practice–based screening for HIV is appealing, given the rising prevalence of the condition in the United Kingdom (especially London), a good prognosis if treated early, the high proportion of cases (24 %) that remain undiagnosed in the community and the high proportion (47 %) of patients diagnosed with advanced disease [26]. The British HIV Association and the National Institute for Health and Care Excellence both support community-based testing in areas where the prevalence of diagnosed HIV is above 2 per 1000 adult population [27, 28], but such testing has not previously been evaluated experimentally in real-world conditions.

Rapid (near-patient) testing provides an accessible means of testing large numbers of people in non-specialist settings. We used the INSTI™ HIV-1/HIV-2 Rapid Antibody Test (bioLytical Laboratories, Richmond, BC, Canada), which is quick to learn and easy to use and thus potentially able to be used by staff with minimal training (see Box 1). The INSTI test has a high sensitivity of 99.6 %. Considering a local prevalence of 2 in 1000 in the United Kingdom, this means that only 1 per 125,000 test results can be expected to be false non-reactive. Owing to the 3-month diagnostic window period, the test may fail to detect HIV in the early, acute phase of infection [29]. Patients with a non-reactive result with no recent risk can be assured of their negative HIV status immediately, whereas those with ‘reactive’ or ‘indeterminate’ results require confirmatory serological testing [30]. Potentially, then, HIV testing (serology or rapid or both) could be incorporated into the New Patient Health Checks that are currently routine in UK general practice [31].

**Table:** 

Box 1 The intervention: rapid HIV testing in general practice	
The ‘hard core’ of the intervention [16] was the INSTI HIV-1/HIV-2 Rapid Antibody Test (bioLytical Laboratories, Richmond, BC, Canada). The single-use test involves 50 μl of finger-prick blood, which was mixed with a sample diluent and poured onto a membrane unit, followed by a drop of developer and clarifying solution. After about 1 min, either one or two blue dots would appear, indicating one of four possible results: non-reactive (one blue dot, negative), reactive (two blue dots, suggesting the presence of HIV antibody in patient serum), indeterminate (e.g., two faint dots or two dots with one displaying a pale centre, suggesting possible early infection) or invalid (none of the above, suggesting test performed wrongly or a faulty test kit). A reactive or indeterminate INSTI test is not definitive; it requires confirmation with a serological test. The test is 99.6 % sensitive and 99.3 % specific [30]. The ‘soft periphery’ of the intervention was how this test was introduced and how it became embedded in the New Patient Health Check and wider organisational routines. As per trial protocol, each practice received a 90-min training session delivered by the research team and a consultant or specialist HIV doctor or nurse, comprising theoretical elements (rationale) and practical ones (rehearsing pre-test and post-test explanations, if possible with a simulated patient, and performing the test on samples under supervision). The practice lead for rapid HIV testing [nurse or health care assistant (HCA)] received an additional training session on study algorithms and quality assurance procedures. Quality control procedures were offered monthly for the first year of the trial and every 3 months in the second. Dedicated codes were installed on practice computers to capture rapid HIV testing as part of the New Patient Health Check or other consultations. Practices received a small one-off payment (£300) plus £10 per test performed, plus free testing kits and support. The New Patient Health Check is used mainly to collect baseline data on health and lifestyle from new registrants by asking standard questions led by computerised prompts. Raising the possibility of HIV to a new patient in a short, largely administrative appointment alters the nature of this appointment. Pre-test counselling is not provided, and there is no preliminary assessment of risk. Staff are encouraged to use standard phrases when explaining the test (and offering the opportunity to opt out), delivering it and giving provisional results. In the case of a ‘reactive’, ‘indeterminate’ or ‘twice invalid’ result, for example, staff were told to ask the patient to wait in the waiting or consultation room until the general practitioner was available to discuss the result with them and arrange confirmatory serology.	

The trial ran for 28 months between 2010 and 2012. Recruitment took place at 40 of 45 general practises practices in a socioeconomically disadvantaged London borough where the baseline prevalence of diagnosed HIV was 8 per 1000 adult population. Practices were randomised to an intervention arm (implementing rapid HIV testing alongside New Patient Health Checks) or a control arm (usual care). The intervention is described in Box 1.

The primary outcome was timeliness of diagnosis on the basis of mean CD4 cell count of all patients newly diagnosed as HIV-positive in general practice, an indicator of stage of diagnosis. Overall, intervention practices offered 11,180 rapid tests, and 44.5 % of these were accepted. In total, 14 tests were reactive, of which 11 were confirmed to be HIV-positive. Serological testing [e.g., opportunistically by general practitioners (GPs) during routine consultations, and through antenatal screening] identified 21 (intervention) and 14 (control) further cases of HIV. Patients identified in intervention practices had higher CD4 counts (that is, were at an earlier stage of infection) than those identified in control practices [24, 25]. Of the patients diagnosed, 79 % were part of identified risk groups (63 % black African origin, 16 % men who have sex with men). All patients identified via rapid testing were successfully transferred to secondary care, and an economic evaluation showed that the intervention is likely to be cost-effective (unpublished data).

Despite the overall success of the trial and the positive result, there was marked variation between the 20 intervention practices in how many tests were offered and, of these, how many were accepted (see the Results section). This raised important questions and provided the impetus for a retrospective process evaluation of why some, but not all, practices were able to assimilate and sustainably implement rapid HIV testing as part of the New Patient Health Check, even though all had agreed to participate in the trial and knew that a goal of the trial was to increase testing.

### The diffusion of innovations model

**Fig. 1:**
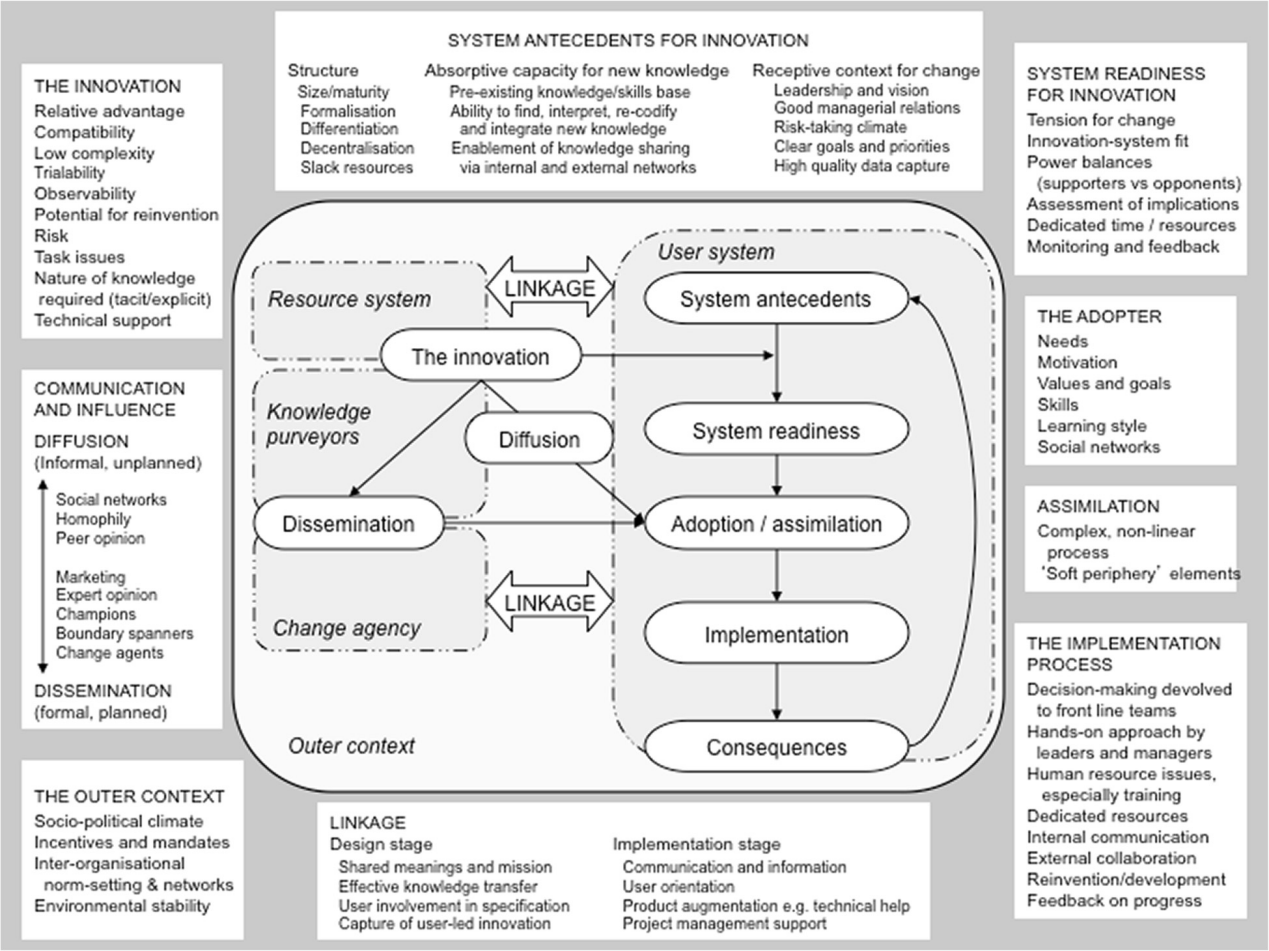
Greenhalgh et al.’s diffusion of innovation model [23]. Figure taken from article by TG in Milbank Quarterly 2004; 82:595. Reproduced under author’s original copyright transfer agreement

A wide-ranging systematic review of the diffusion, spread and sustainability of innovations in the organisation and delivery of health services identified six interacting components: (1) the innovation itself; (2) the intended adopters; (3) communication and influence; (4) the inner organisational or system context, comprising general antecedents for innovation-specific readiness for a particular innovation; (5) the outer (inter-organisational and environmental) context; and (6) the implementation process. The model (Fig. 1) emphasises the importance of linkage between different components of and feedback regarding the consequences of innovation to other parts of the system. The components of the model are defined in Table 1.

**Table 1:** Definitions of components of Greenhalgh et al.’s diffusion of innovations model

Component	Definition	
Attributes of the innovation	How the potential adopter views the pros and cons of the innovation	
	*Relative advantage*	A clear, unambiguous advantage in terms of either effectiveness or cost effectiveness.
	*Compatibility*	Compatible with the values, norms and perceived needs or intended adopters.
	*Low complexity*	Composed of simple, easy to implement steps; able to be broken down and learned on an incremental basis.
	*Trialability*	Can be experimented with.
	*Observability*	Benefits are (or quickly become) visible to intended adopters.
	*Potential for reinvention*	Possibility to adapt, refine or otherwise modify the innovation to suit adopter needs.
	*Fuzzy boundaries*	If innovations have ‘hard cores’ (irreducible elements of the innovation) and ‘soft peripheries’ (structures and systems required for full implementation), adaptation of the soft periphery can facilitate adoption.
	*Risk*	Risks of the innovation (as perceived by the intended adopter) are outweighed by its perceived benefits.
	*Task issues*	Extent to which the innovation is relevant, feasible, workable and easy to use for the adopter.
	*Nature of knowledge*	Knowledge required to enact the innovation can be transferred, either by codification (explicit knowledge) or more informally, e.g., by shadowing (tacit knowledge).
	*Technical support*	If the innovation is technical, help desk support is available, especially in the early stages of implementation.
System antecedents for innovation (including structure and/or absorptive capacity and/or receptive context)	Extent to which the organisation is ready for innovations in general	
Structure	*Size and/or maturity*	Practice size is related to innovation adoption, with larger practices faring better regarding implementation; a proxy for other features, e.g., slack resources and functional differentiation.
	*Formalisation*	The extent to which there are rules and protocols regarding organisational activities which are upheld.
	*Differentiation*	The extent to which roles and activities are divided.
	*Decentralisation*	Decision-making power is appropriately dispersed across organisations.
	*Slack resources*	The resources an organisation has beyond what it minimally requires to maintain operations.
Absorptive capacity for new knowledge	A dynamic capability pertaining to knowledge creation and use that enhances an organisation’s ability to gain and sustain a competitive advantage.	
	*Pre-existing knowledge and/or skill set*	Existing knowledge and skills within the organisation; particularly facilitatory if somehow related to the innovation.
	*Ability to find, interpret, recodify and integrate new knowledge*	The ability to take on, understand, integrate into existing systems and put into productive use new information.
	*Enablement of knowledge sharing via internal and external networks*	Individuals are able to share knowledge regarding the innovation internally and externally through established networks.
Receptive context for change	A combination of factors from both the inner and the outer contexts that together determine an organisation’s ability to respond effectively and purposefully to change.	
	*Leadership and vision*	Top management support, advocacy of the implementation process and continued commitment to it enhance the success of implementation and routinisation.
	*Good managerial relations*	Staff have positive relationships with managers.
	*Risk-taking climate*	A supportive working culture where practice staff feel able to experiment with new innovations without fear of being reprimanded.
	*Clear goals and priorities*	Objectives are clear to the organisation and the staff.
	*High-quality data capture*	Organisational systems are in place to obtain high-quality data related to the innovation diffusion.
System readiness for innovation	The extent to which the organisation is ready for the specific innovation.	
	*Tension for change*	Degree to which adopters see the current situation as inadequate or intolerable.
	*Innovation system fit*	The innovation fits with existing values, norms, strategies, goals, skill mix, supporting technologies and ways of working within the organisation.
	*Power balances*	
	*Assessment of implications*	The implications of adoption are known and assessed.
	*Dedicate time and/or resources*	Degree to which budget and resources available are adequate and recurrent.
	*Monitoring and feedback*	Systems and skills are in place to monitor and evaluate the impact of the innovation and feedback to adopters.
Adopter	Those meant to adopt and enact innovations.	
	*Needs*	What the adopter needs to be able to adopt the innovation.
	*Motivation*	Whether the adopter is motivated to adopt the innovation.
	*Values and goals*	Does the innovation gel with the adopter’s values and goals?
	*Skills*	The skills required to adopt the innovation and whether adopters possess these.
	*Learning style*	The ways that adopters learn are considered and catered to in the innovation training.
	*Social networks*	The patterns of friendship, advice, communication and support that exist among members of a social system.
Implementation process	The process by which a new innovation is diffused across an organisation.	
	*Decision making devolved to front-line teams*	Do lead users of the innovation have control over aspects of the implementation process?
	*Hands-on approach by leaders and managers*	Leaders and managers are involved in the implementation process, supporting and assisting problem solving as required.
	*Human resources issues, especially training*	Have all human resources issues linked to the introduction of the innovation (training, workload, supervision, performance management) been addressed adequately?
	*Dedicated resources*	Specific resources of time, budget and other relevant resource are dedicated to support implementation.
	*Internal communication*	Involved bodies communicate effectively with each other regarding the innovation and the implementation process.
	*External collaboration*	Effective knowledge-sharing links to other organisations who are implementing the same innovation.
	*Reinvention and/or development*	Was it possible to adapt the innovation or the tasks and processes associated with it to suit local contingencies?
	*Feedback on progress*	Are there evaluative and feedback mechanisms in place and enacted?

Applying this model to a RCT design is not straightforward, because the evidence on which it is based relates to free-living individuals operating in real-world conditions. In particular, the element relating to communication and influence was less relevant to this evaluation, because all practices and participating staff received a standardised training package (Box 1). Nevertheless, the pragmatic design of the trial meant that many real-world influences were built into the study design. For example, participating practices were open to communication from other practices locally as well as from other, ‘outer context’ influences, such as the economic recession, new immigration and changes in national and local HIV policies.

The aspects of the model that were most relevant to the process evaluation were staff perceptions about the intervention (testing was undertaken by practice staff, who had different views about the value and appropriateness of the test and their own role in it), the organisational antecedents and readiness for innovation as well as the implementation and assimilation process.

## Methods

### Management and governance

Full details of study management and governance, including the independent data monitoring committee, are given in the main empirical report [24, 25]. The trial (ISRCTN63473710) was approved by Camden and Islington Community Research Ethics Committee (09/H0722/67). Ethical approval for the qualitative research was gained from Bloomsbury National Research Ethics Service committee (11/LO/0324) in April 2011 with an amendment in December 2013.

### Data sources for process evaluation

Various methodologies and data sources were used.

#### Participant observation

Throughout the trial period, HM was a member of the study team responsible for practice recruitment, training, monitoring and general liaison. This work required her to make frequent visits to practices, which were typed up formally as field notes as soon as was practicable after each visit. Numerous informal conversations and email exchanges also took place with practice staff regarding all aspects of implementation, including the thoughts and feelings of front-line staff about HIV testing and their narratives of test enactment. This ‘autoethnographic’ approach is widely used in organisational case study research and can provide a particularly rich account of organisational culture and practices [32, 33].

#### Qualitative interviews

Semi-structured interviews were undertaken with a purposive sample of 21 staff in 16 of the 20 intervention practices; the other four practices failed to respond to requests. Most were nurses (*n* = 11) or HCAs (*n* = 7) who primarily offered the rapid HIV test as a part of the New Patient Health Check. One practice manager, one clinical manager and one GP were interviewed in relation to their role in rapid testing (e.g., managing patients with reactive or indeterminate rapid test results, overall coordination of testing within the practice). Interviews were conducted at the practice during normal working hours and were one-to-one, except for two nurse and HCA pairs who asked to be interviewed together. Written informed consent was obtained from all participants, who also completed a short demographic survey regarding age, ethnicity, length of time at current practice, part-time or full-time employment and previous HIV-related experience. Interviews lasted between 30 and 60 min. Interviews were conducted throughout the final 8 months of the trial and into the following year. Participants were given a £10 voucher as compensation for their time.

Proust et al., in a feasibility and acceptability pilot study published prior to the trial, reported on patient views regarding rapid HIV testing in general practice [34]. Qualitative interviews with patients offered a rapid test as a part of the New Patient Health Check found that patients found the offer of a test acceptable and that they found the reduced wait time for results and the accessibility of testing to be appealing. Concerns included a possible lack of support for the newly diagnosed patient and patient preparation for testing [34]. Interviews are currently being undertaken with consenting patients who were diagnosed as HIV-positive through the trial and rapid testing. In a forthcoming article, we will report on patient diagnostic experiences of rapid HIV testing as part of the New Patient Health Check in primary care.

#### Trial performance at practice level

Practice-level performance data were collected through the remotely accessible electronic record systems used in participating practices (EMIS [35] and VISION [36]). This allowed the research team to gather regular data on the number of rapid HIV tests offered, performed and declined at each practice. Upon completion of the trial, data were aggregated and overall trial performance was analysed. In addition, the number of HIV serological tests per practice (i.e., tests sent to the hospital laboratory either to confirm a rapid test result or for other clinical reasons) was compiled quarterly.

More generally, practice demographic data (including practice list size, index of multiple deprivation score, level of male serological HIV testing prior to the trial) were collated to enrich the case study and inform the application of the diffusion of innovations model.

### Data analysis and case study construction

Data analysis occurred in two phases: (1) preliminary familiarisation and coding and (2) synthesis into case studies. In the preliminary phase, qualitative transcripts (field notes, interviews and extracts from emails and documents) and matched demographic data on interviewees were uploaded into NVivo software (QSR International, Doncaster, Australia) and framework analysis was undertaken [37]. Selected transcripts (sampled for diversity and richness) were used to develop a preliminary coding frame. This framework was then applied to all transcripts, with emerging themes noted. Coding reports were generated. This process was applied twice. The first time it was designed to organise and gain familiarity with the data using the question, What were the experiences and perspectives of providers of rapid HIV tests in primary care? The second time it was used to bring in the components of the diffusion of innovation model (Fig. 1) to consider the question, What enabled or hindered providers in effectively implementing rapid HIV testing in general practice? After producing preliminary categories, we iteratively refined these in team discussions using the constant comparative method—that is, comparing each new item of data with an emerging picture of the case as a whole [38].

We created a spreadsheet of practice characteristics that included practice size, male HIV testing rate prior to the trial, rapid HIV and serological testing and practice HIV diagnoses during the trial period. One striking (and initially surprising) finding was that practices that had had high rates of male serological testing for HIV before the trial (a proxy for the level of prior awareness and interest in HIV in that practice) were not always high performers in rapid testing. This analysis informed the sampling of four contrasting case studies to help theorise the findings using diffusion of innovations:

- Practice A: high serological testing, high rapid testing
- Practice B: low serological testing, high rapid testing
- Practice C: low serological testing, low rapid testing
- Practice D: high serological testing, low rapid testing

Our aim in constructing the case studies was to produce a rich and meaningful account of how and to what extent the rapid HIV testing intervention was assimilated and implemented in each participating practice, all of whom showed enthusiasm for adopting the intervention. We used team discussions and applied narrative as a sense-making and synthesis tool to weave together the quantitative and qualitative findings for that practice into a rich picture that depicted key perspectives, events and upstream causes while also conveying ambiguities and uncertainties [39]. In this way, the strengths and weaknesses of each practice for the purposes of implementing the intervention were revealed and explored. We sent drafts of our interpretation to practices who were interested in seeing them before finalising the interpretations presented below.

## Results

### Description of dataset and introduction to case examples

The final dataset for the process evaluation comprised 60 pages of field notes, 245 pages of interview transcripts and 70 pages of additional free-text documentation, plus quantitative data on the distribution of 11,000 rapid HIV tests across 20 intervention practices and 5193 serological (hospital laboratory) tests across 40 intervention and control practices, respectively.

### Common findings: relative advantage and simplicity of the rapid test

Despite wide variation in uptake of rapid testing between practices, there were some findings common to all, particularly in relation to the intervention (Box 1). The front-line staff who delivered the intervention almost universally perceived a distinct relative advantage (and considered that patients also saw an advantage) in rapid, accessible and convenient testing in general practice compared with usual care (the serological test requiring venepuncture and at least a 2-day wait for results). The quick and actionable results would mean less waiting and administration and, many staff believed, fewer losses to follow-up. Staff reported that patients appreciated receiving their results instantly, and they themselves gained satisfaction in being able to provide this information quickly.

Staff and patients felt that placing the rapid HIV test within the New Patient Health Check with an ‘opt-out’ option allowed people with low awareness of HIV and low concerns about testing to access a test easily, thereby extending the reach of testing.

*Interviewer: Do you think it’s a good idea to test it in that way?*

*HCA: Yes, 100 %.*

*Interviewer: How come?*

*HCA: Because most people don’t even think about it at all. They could go on their whole lives not thinking about it and people are quite—I don’t know if ‘ignorant’ is the right word to use. If you offer somebody at a consultation on a one-on-one an HIV test, they might get a bit offended. But this way, if you’re saying it’s something that we’re doing at this point in our practice, as a new patient joining us it’s offered randomly, it just gives people a chance to think about if they do want it. If they decline, then at least they can come back and say, “You know, I was offered this test, and yes, I would like to have it done.” –***HCA from practice D**

Lack of need for pre-test and post-test counselling and detailed sexual history testing, as well as location of the test in the context of a routine general practice encounter, effectively normalised and destigmatised the rapid HIV test and made it relatively easy for non-specialist staff to learn and deliver (and for patients to accept). However, HCAs in particular do not routinely test for what is considered a stigmatised and serious condition, so the test did require some change in their role and the way they related to patients—an issue that played out differently with different staff and in different practices (see case studies below).

Staff also commented that patients preferred rapid finger-prick testing to venous blood sampling. The test was technically simple, and phlebotomy skills were not needed. Even patients who disliked needles did not seem to mind the small lancet used quickly in the rapid test, a finding we demonstrated previously in a pilot study [35]. They also said that patients preferred the near-patient test, as they could visibly see that the result was their own, thereby increasing their trust in the test result.

In sum, the INSTI HIV-1/HIV-2 Rapid Antibody Test (the ‘hard core’ of the intervention; see Box 1) was perceived extremely positively by the staff charged with delivering it. Below, we present four contrasting case studies of practices where different individual and organisational factors combined to produce four very different contexts for assimilating, implementing and sustaining the intervention for the duration of the trial.

### Practice A (high recruiter): high system antecedents, high system readiness

Practice A implemented the rapid testing intervention very successfully, offering more rapid tests than any other practiceand having a moderate decline rate (42 %), though only one case of HIV was detected via the New Patient Health Check. Our qualitative and quantitative data showed that effective implementation of the test was the result of key system antecedents for innovation, high system readiness for the rapid test and a smooth implementation process and strong adopter factors among front-line staff (see Fig. 1).

Practice A was one of the largest practices in the borough. It was mature and well organised, with a clear differentiation of functions and staff roles and good managerial relations. For example, the practice nurse and HCA had been with the practice for some time. They felt their roles were clear, and they understood who should be called upon and at what stage if a test was reactive. Both expressed the importance of GPs in making diagnoses, both for the patient and for the sake of their own comfort in offering tests. If needed, they sought information and clarification from senior staff.

*I’ve had a couple of patients say that they didn’t want the test at the time I offered it, in the New Patient Health Check, but is it okay if I go away, think about it and then maybe come back? And I’ve said, Well, you know, this is something that we offer now. If you come back, then I’d have to question that with the doctor as to whether you can have it as a, you know, fully registered patient. I’ve spoken, I did speak to a doctor actually, and they said that it would be okay if they hadn’t been registered too far down the line. –***HCA from practice A**

Junior practice staff were mentored by more senior staff, providing both pastoral support and opportunities for individual and team learning (the latter linked to the key construct of absorptive capacity; see Table 1). The practice was able to integrate new knowledge through regular practice meetings and feedback. Practice A showed interest in the monitoring of progress and the study’s overall performance, often asking how they rated in relation to other trial practices.

Leadership, organisation and communication appeared to be strong factors in practice A. For example, a lead was assigned for the intervention and provided support to junior staff tasked with delivery. Roles were well differentiated, and support was provided promptly when required.

*Interviewer: But you’ve had a reactive?*

*Nurse: That was an early one.*

*HCA: Yeah.*

*Nurse: Trying to; I’m trying to recall it.*

*Interviewer: Okay.*

*Nurse: As to what, as to what I actually said. I remember I sent a screen message to (GP A), and I, I think I just said something like, oh, that I needed the doctor to verify the result and that I needed him to look at it. I think it was something like, that, it’s such a long time ago now, and then (GP B) came in, and I had a chat with him, and we did the blood test, gave him some information, and I think (GP A) said that he would be in contact with him. –***Nurse and HCA from practice A**

Staff in practice A perceived the intervention positively and were also proud of the overall quality of service they offered. They viewed the new test as enhancing that quality.

*Nurse: Yeah, I think, the impression I get is that they think that we’re been quite thorough and that we’re, you know, so I think it, I think it promotes us.*

*HCA: That we’re very organised, well, she said I’m very organised and thorough.*

*Nurse: Yeah, that we care and that we’re offering a good service. –***Nurse and HCA from practice A**

Perhaps partly for this reason, rapid testing was quickly incorporated into the New Patient Health Check and was viewed by staff as a good fit with that process (a construct described in the literature as ‘innovation–system fit’ [23]). Practice A was also one of the few practices that did not stress time constraints (linking with what in the model is called ‘slack resources’, defined in Table 1).

Early in the trial, a positive HIV diagnosis through rapid testing was made, demonstrating that the innovation ‘worked’ and achieved its objective, an attribute known as *observability*. This is likely to have reinforced the implementation process (see feedback arrows in Fig. 1).

In sum, practice A illustrated many of the key organisational preconditions for successful assimilation of innovation, including key elements of structure (large practice list size, maturity, slack resources, functional differentiation), absorptive capacity for new knowledge (high pre-existing knowledge and skills base and formal and informal processes for knowledge sharing among staff from different professional groups) and high readiness to change (leadership and vision, good managerial relations, risk-taking climate and high-quality data capture). It also showed high readiness for the particular innovation (innovation–system fit) because clinicians were already interested in HIV testing and keen to promote it further. Importantly, nobody in the practice appeared opposed to the innovation.

### Practice B (high recruiter): moderate system antecedents, exceptional front-line staff, strong internal synergies

Practice B also assimilated rapid HIV testing very effectively as part of the New Patient Health Check. Despite being one of the small to medium-size practices and having a low turnover of patients (and hence fewer new registrants), this practice diagnosed twice as many patients through rapid HIV testing as any other practice in the study. The number of rapid tests offered (*n* = 870) was high for practice size, and the rate of tests declined was low (36 %). Yet, the serological testing rate prior to and throughout the trial was fairly low [fourth amongst the 40 (0.66/1000 serological testing rate during the trial period, and 2.07/1000 prior to the trial, respectively) participating practices prior to the trial], suggesting that the practice did not previously place significant emphasis on HIV testing. A number of factors at both the organisational and individual levels may help explain this success.

Practice B demonstrated moderate system antecedents and readiness for innovation (see Fig. 1)*.* The practice was well organised and had a clear and harmonious differentiation of roles; the nurse spoke highly of senior doctors and vice versa. The practice also had high absorptive capacity for new knowledge and a receptive context for change. This existing knowledge and willingness to learn more also point to the practice’s goals and priorities of supporting patients beyond immediate medical needs. On one occasion when there was concern about misinterpreted results, the nurse immediately discussed next steps with the GP and ensured the safety of the patient. The good managerial relationships and strong communication shown here may also indicate a risk-taking climate in which interacting with innovations is encouraged and solutions to any challenges are found together when needed.

*Respondent: There was one which did … that was indeterminate. There was … you know, the pots. It was … it was supposed to be non-reactive, but inside that pot it was like a line.*

*Interviewer: Okay. Right. Just a straight line.*

*Respondent: And when I told the doctor, he say, probably … no, not the doctor; the lady that came the other day. He said probably it is damaged or something like that. But I told Doctor A, and he said I should call the patient back, you know. So, we call the patient back, and I explain, even to the patient as well, that this result, it doesn’t mean you have HIV now, but it might be one thing or the other that is making the … you know, the test to being invalid. So … and she decided … she came back.*

*Interviewer: Had another test.*

*Respondent: Yes. And it was non-reactive. –***Nurse from practice B**

Although the practice had low serology rates prior to the trial, once testing was introduced and the staff were trained, the intervention was quickly adopted. Staff appeared engaged, seeing the relative advantage of the innovation.

An unusual feature of practice B was that a single individual (the practice nurse) undertook all New Patient Health Checks, for which she had a generous time allocation (30 min for each). She worked full-time and had her own dedicated consultation room. She had a professional and strongly patient-centred approach to her job, working largely autonomously and indicating general enjoyment of what she did.

The nurse who did all the rapid testing framed it not merely as a service for individual patients but also as an ethical imperative and a way to improve public health; in other words, it had particularly high value and significance for her as a professional.

*I think I just like doing it because it is good. When you think about the end result, is good. It makes you feel you have done something good as well. At least for somebody who doesn’t know that is positive and is not, because although the news of being positive, it has a lot of effect on them, but after counselling…. But I believe it will prevent other people as well, or protect other people. Either prevent or protect from catching it because if it is known, then the patient can take precaution not to infect other people. –***Nurse from practice B**

Quality control visits showed that the lead nurse for rapid testing, along with other practice staff, managed to ‘reinvent’ the test and the algorithm to suit local practice conditions without losing fidelity. The nurse felt concerned at the potential effect of a reactive result on the patient in the room, so the nurse began to perform the definitive aspect of the test away from the patient’s view—an adaptation that was not in the original training. She did not disclose to the patient that the test took 1 min, allowing herself a few moments when required to reflect on test results and plan her next steps.

*Interviewer: Yes. How did you feel the first time you saw a reactive?*

*Nurse: I was … but I was looking, but he wasn’t looking at me.*

*Interviewer: Yes, because you do it on that side of the room.*

*Nurse: Yes. On that side. So he was sitting down there, so … but he was looking at me as well. But because I was facing that side, he couldn’t see my face. –***Nurse from practice B**

Another adaptation in practice B was that GPs would refer patients to this nurse for rapid testing, regardless of whether they were booked for a new patient check. The nurse reported that some patients for whom the possibility of HIV infection was being considered were persuaded to have the rapid test when they may have declined the more invasive and less convenient serological testing.

As in practice A, a positive HIV diagnosis through rapid testing was made early in the trial, reinforcing staff confidence in the test.

Practice B is noteworthy, not merely for possessing many (though not all) key system antecedents and readiness factors for innovation and highly motivated front-line staff, but also in the way these elements were combined. The very professional and patient-centred practice nurse, for example, was able to give her very best to the study because the practice allocated plenty of time and allowed the nurse to work independently and adapt the innovation to suit her own working style and local microroutines. More subtly, the culture of the practice was to embrace innovations and support their embedding. Doctors recognised the nurse’s competence and interest in this innovation and began to send her additional patients for testing. In these and numerous other ways, the elements of innovativeness built on one another synergistically.

### Practice C (low recruiter): low system antecedents, reluctant front-line staff

Practice C struggled to implement rapid testing. The practice was slow to offer the first test, and its rate of testing remained low throughout the study (in total, 72 rapid tests were offered, and 50 % of these were declined), despite multiple visits and ‘retraining’ from the research team. It had a low serology HIV testing rate prior to and throughout the trial. Low recruitment from this practice was explained by a combination of factors, both organisational and individual.

System antecedents were low in practice C. A small practice, it comprised three GPs, one nurse and one HCA (both of whom undertook New Patient Health Checks), one practice manager and two receptionists. Located within a large building housing multiple practices, the surgery; always seemed crowded and very busy.

The practice showed little interest in, or time to accommodate, other innovations, and there were few resources (human or financial) available to invest in new projects. Overall, the practice appeared to find a new service model difficult to integrate into business as usual. There was expressed frustration with changing National Health Service (NHS) policy and guidance as well as broader changes in health care culture. A low absorptive capacity for new knowledge was also evident. One of the doctors, for example, asked the research team how to access information and register for GP training courses unrelated to the intervention, suggesting that this individual found locating and navigating information difficult. Significantly, practice staff did not perceive a great need for HIV testing in the borough, suggesting that there was little, if any, tension for change. The nurse described herself as ‘overstretched’. She gave the impression of barely being able to complete her existing work and having almost no personal capacity for additional tasks:

*[The rapid HIV test] really is not a problem. It’s just, you know, having the time. I mean, often I get to the end of a morning, and I feel like a rag. –***Nurse, practice C**

Because of the understandable reluctance of busy front-line staff to accommodate the test, it never became routinised within the New Patient Health Check in practice C; it was not offered to most patients having these checks, and, unlike in practices A and B, it never came to be viewed by staff as *part of* that check. With such low numbers of tests being undertaken, it was not surprising that no cases of HIV were detected using rapid testing, so its observability was not evident in this practice.

Our data suggest that there may also have been an issue about the compatibility of the test with the values of the HCA, who appeared personally uncomfortable testing for HIV. Indeed, it is unclear whether this staff member offered any tests throughout the trial period. This was a source of frustration to the nurse, who had tried to rectify the situation:

*I don’t have any problem with doing [the rapid HIV test]; the actual doing of the tests is straightforward. My colleague who should be doing them as well hasn’t done one. I don’t know. I went through it with her again a while ago; I don’t know, two or three weeks back I went through it again with her to remind her how to do it. And I do it whenever I can, but my problem is time…. I don’t know if it’s a religious thing, maybe [explanation of perceived religious views of colleague]. I don’t know if it’s something to do with that. But she’s a health care assistant; she’s not a nurse. That’s a difference as well. –***Nurse, practice C**

The nurse raises an important point here—that the rapid HIV test was not merely a technical procedure but a professional interaction. Technically, it was simple and straightforward (albeit hard to accommodate if time was short), but because of its link to a stigmatising illness, it also required a professional, rather than merely transactional and task-oriented, relationship with the patient. Implicitly, the block to adoption may not have been the HCA’s views per se but the fact that her role—in this practice, at least—was not professionalised. HIV remains a stigmatised condition, and the line between a screening test and a diagnostic test can be fine, particularly in the case of the test used in the trial, which may be interpreted by patients as well as providers (two dots as a reactive result, one dot as a non-reactive result). It may have been that reluctance to offer rapid testing relates to the need to provide immediate feedback regarding test results. Whereas GPs are called upon to share test reactive results, HCAs and nurses expressed significant concern about managing reactive results and patient reactions as well as the interval between the test and calling upon the GP. This may have been a factor in the HCA’s reluctance to test. The nurse, though personally motivated and more professionally experienced, had only limited opportunity to offer rapid HIV testing, as most New Patient Health Checks were performed by the HCA.

It is also significant in the quotation above that the nurse took personal responsibility for trying to change the HCA’s attitude and behaviour in relation to rapid testing. Despite raising the issue with GPs and the practice manager, no action was apparently taken to explore or improve this staff member’s low performance on trial activities. In contrast to the subtle but important involvement of senior clinicians and managerial staff in practices A and B, the approach of similar staff in practice C was distinctly ‘hands off’.

It is noteworthy that the practice nurse made numerous efforts to implement the rapid test, but those efforts had very limited success in the context described above. For example, she showed creativity in ‘reinventing’ the finger-prick aspect of the test. (“*As long as I get a decent drop of blood, just occasionally people don’t bleed terribly well. I don’t like the finger-pricker they give with it. I tend to use my ones…. They’re a bit more gentle.”*) This motivation and creativity did not translate into tests actually performed, however, because most New Patient Health Checks were done by someone else, and the low absorptive capacity of the practice meant that the nurse’s improved method of testing was not effectively shared with the front-line staff member who had the most opportunity to actually do the test.

In sum, practice C was not an innovative practice, nor was it ready for the specific innovation of rapid HIV testing. The member of staff on which the intervention most depended was personally reluctant, and factors known to help the implementation phase (notably hands-on input from senior staff) were absent. In this environment, the presence of a single, keen and committed member of staff had only limited impact on the implementation of the intervention.

### Practice D (low recruiter): keen doctors but low system antecedents and negative synergies

Practice D also struggled to implement rapid HIV testing as a part of the New Patient Health Check. The 557 rapid tests that were offered during the trial period (of which 43 % were declined) may appear relatively high, but the size of the practice and consistent registration of new patients demonstrated a number of missed opportunities for testing. The pattern of testing over time suggests that the innovation was never effectively routinised. Rather, periods with very low rapid testing were interspersed with periods in which a number of tests were performed within a short period of time.

On the surface, this low recruitment rate was surprising. Several of the GPs had a clinical interest in HIV; HIV serological testing rates were high both before the trial and during it (696 performed); and a high turnover of patients ensured high numbers of New Patient Health Checks.

As one of the largest and most diverse practices in the borough, practice D comprised 15 GPs, 9 nurses, 3 HCAs, 2 practice managers and more than 10 receptionists and administrators. Many staff worked part-time. There was time pressure on many activities, and the practice was constantly busy. The striking contrast between the very high HIV serology rates but very low rapid testing rates may be related to our finding that there were two distinct work cultures within the practice. Many of the GPs were highly qualified with some involved in community-based projects. Others had an interest in sexual health and regularly offered regularly offered opportunistic regularly offered opportunistic serology testing for HIV.

However, the nurses and HCAs appeared to have little or no involvement in these activities or protected time to become involved. Knowledge appeared to circulate well among the doctors, but to a much more limited extent between the doctors and the other practice staff, suggesting a problem with absorptive capacity (see the Discussion section). In general, non-medical staff did not have academic links. Many worked part-time and had a very task-oriented attitude toward their work (i.e., they came to work, completed what was expected of them and went home). Some staff described a lack of harmony in practice relationships as well as a sense of being personally overstretched. There appeared to be relational tensions between some staff in the practice that affected the implementation of the study protocol. For example, HCAs had asked reception staff to hand out leaflets about the study to patients at the reception desk, but this did not always happen. Unusually, the research team assisted in mediating this issue.

Although front-line staff expressed enthusiasm about providing testing and acknowledged the value of offering the test, they also viewed involvement in the trial as an additional task in their already high workload. The doctors in practice D viewed involvement in this trial as important both for them as professionals and for the practice population, but they did not appear to discuss with front-line staff how the innovation could successfully be incorporated into an already busy practice. As a result, opponents of the innovation (‘yet another task’) outnumbered supporters, and because it was nurses and HCAs who actually delivered the intervention, these individuals were more strategically placed to do so. Bursts of trial activity probably reflected periodic encouragement of front-line staff by GPs concerned to increase the practice’s performance statistics, but this is very different from *routinising* the innovation as business as usual (see the Discussion section).

Although decision making about offering the rapid test was largely devolved to front-line teams, this was complicated by poor communication and strained relationships, to the extent that front-line staff did not appear inclined to take responsibility for implementation. There was also a significant problem with time and resources because HCAs were often called upon to refocus their work for short periods to meet particular practice goals. There was little inter-practice feedback unless it was prompted by the study team, minimising opportunities for creating the kind of positive feedback loops that were evident in practices A and B.

These organisation-level factors significantly overshadowed other, more positive elements of this practice in relation to HIV testing, including the perceived relative advantage of the rapid test in comparison with the widely used serological testing, and the compatibility of the test with the values and goals of the practice. In addition, whilst most front-line staff found the test simple and easy to use, one HCA (unusually) reported struggles with the material aspects of the test and indicated that, on some occasions, this stopped her from offering testing. Even HCAs who expressed strong enthusiasm for testing felt they were often unable to offer tests, however, owing to a lack of time as well as a lack of continuity in their role.

*But because it was coming up to the end of the financial year and everyone had to tally up QOF points for diabetes and these and this and that, it took priority. If people had come in, obviously if there were new patients, we wouldn’t turn anybody away, but we were phoning up and pre-booking patients to come in for their diabs or their foot checks or their blood pressure. And because I’m only now doing 3 days a week, I literally split sessions between here and (another practice). I do here three sessions and there three sessions. So, when I am here, they get me to do loads of ECGs and different other things, and then when I’m there, I’m doing things over there that they need doing. –***HCA, practice D**

Moreover, despite a number of HIV diagnoses made using serological testing, no diagnoses were made using rapid tests, indicating a lack of observability*.* It is telling that, whereas doctors in practice B altered their behaviour during the trial by sending patients to the nurse for rapid HIV testing, those in practice D continued to use serological testing when they suspected possible HIV in a patient. It appears that the rapid testing was seen as the province of a different group of staff, not something that was business as usual. GPs become involved in the rapid HIV testing algorithm in cases of reactive, indeterminate or invalid results, but because none occurred at practice D, this may have impacted their knowledge and involvement in trial activities.

In sum, despite much initial enthusiasm, practice D was impeded by a combination of structural, capacity-related and cultural factors (most crucially, limited slack resources), along with individual adopter traits and a weak process of implementation.

## Discussion

### Summary of findings

This process evaluation of a complex intervention trial in UK general practice has demonstrated the usefulness of the diffusion of innovations model in explaining variation in performance of participating practices. In particular, five aspects of the model appeared to distinguish high-performing practices from low-performing ones.

#### System antecedents for innovation

Larger, more formally organised practices with an appropriate division of roles and slack resources (especially time), as well as those with strong communication networks and good managerial relations, were higher recruiters.

#### System readiness for the innovation

Practices with well-organised New Patient Health Checks, clear and stable staff roles for these checks, that had many supporters of rapid HIV testing and that were able to dedicate time and resources to incorporating the test smoothly into practice routines were better able to implement testing.

#### Adopter characteristics

Staff who perceived the test to be beneficial to patients, easy to undertake and professionally meaningful undertook more tests.

#### The implementation process

Uptake of the intervention was smoother and more likely when both senior clinicians and managers took a hands-on approach. If practices devolved decision making to front-line teams but did not follow up with support and feedback, implementation suffered. Dedicated resources such as time, space and support for implementation appeared critical.

#### Reinvention and local customisation

Small adaptations to how, where and by whom the test was conducted, without losing fidelity of the core components, sometimes appeared to make a significant difference to its acceptance and routinisation within the practice, though reinvention alone sometimes failed to overcome wider structural or cultural barriers.

Despite the good fit between individual components of the model and our case study data, it is important to stress that our findings also illustrate how these components may (but do not always) act synergistically and interact dynamically, allowing strengths in one component to compensate for limitations in another. Conversely, the presence of individual elements conducive to innovation does not guarantee success, since the overall practice dynamic may prevent particular factors from having a positive influence. This is important because it means that, whilst all the elements described above are ‘evidence based’, the way they play out in any particular organisation will be hard to predict.

Additionally, as acknowledged by Greenhalgh et al. in their discussion of the diffusion of innovations, greater consideration of the transferable lessons from cognitive and social psychology is needed [23]. Models of innovation diffusion are based largely on a dyadic interaction between a single adopter and an intervention. Rapid testing in primary care produces a triad between the provider (the adopter), the rapid test (the innovation) and the patient. We found that in many cases the views and actions of providers depended on their assumptions about patient feelings and reactions to the offer of a rapid HIV test.

*When we did the training, we were sort of told, with the reactive result, you are to leave the room and get a doctor. I haven’t had to do that yet, but I don’t know how that would make the patient feel, if I am just getting up and walking out…. I mean, I don’t think it was as abrupt as all that in the training…. I don’t know how people feel about that, but obviously something is going on…. Would I just make them more nervous? –***HCA**

Unsurprisingly, this provider ‘theory of mind’ regarding the patient entered the calculus of offering testing and was a strong aspect of the discussion of the innovation. We feel this is an underdeveloped aspect of the diffusion of innovations model which precludes a more nuanced discussion of the health care consultation, the role of the patient and the impact of new innovations within it.

### Implications for involving organisations in complex intervention research

Our findings support the conclusion that there is not, nor can there ever be, a universal implementation model for complex interventions. Site-specific characteristics and realities need to be considered. Complex interventions, such as other service-level innovations, cannot be treated as ‘bolt-ons’, but must instead be carefully integrated with practice systems to become part of business as usual. This process is known as ‘routinisation’. An organisational routine is a recurrent, collective, interactive behaviour pattern implemented (often largely subconsciously) by individual actors through shared knowledge and practice [40]. Routines are path-dependent; that is, they are shaped by historical particularities in any given setting, so there is no such thing as universal best practice. Whilst routines confer stability in an organisation by conveying a strong sense of what is ‘business as usual’, they also contain within them the seeds of change because they depend for their enactment on here-and-now decisions by individual actors whose creativity can allow a change to the routine (and hence ‘reinvention’ of the complex intervention).

Practices who were successful in implementing the rapid HIV test as part of the trial had routinised the innovation not merely by assigning its component tasks to particular staff members but also by encouraging and rewarding those staff for embedding it in the day-to-day work of the practice and linking it to other routines. This crucial distinction between ‘complex intervention as a set of tasks’ and ‘complex intervention as embedded routine’ aligns with Denis et al.’s notion of the ‘hard core’ of a complex intervention (the elements that constitute its ‘fidelity’) and the ‘soft periphery’ which can and must adapt to accommodate it [16]. In cluster randomised trials, the unit of intervention is large (an entire organisation, as opposed to individual participants), so poor uptake of the intervention by one participating unit can significantly threaten the success of the trial [5].

We cautiously conclude that one way in which researchers might guard against such eventualities would be to meet with practices prior to recruitment and use the diffusion of innovation framework to consider the different ‘soft periphery’ aspects for the proposed intervention. General practices are diverse spaces and vary widely even within a small geographic locality. Much may be gained from highlighting the practice’s strengths and weaknesses in relation to a particular innovation (‘assessment of implications’ in Fig. 1).

Such an assessment should include, for example, consideration of what is acceptable research ‘fidelity’. Pragmatic trials are meant to account for the contextual factors implicated in the ‘real-life’ settings where trials are conducted [6]. In diverse settings such as general practice, greater consideration of how we define research fidelity may be required. As discussed by Hawe and Shiell, ‘fidelity defined functionally rather than compositionally’ may be key [15]. The point is to allow the interventions to be responsive to their context while still being meaningfully evaluated.

Perhaps drafting site-specific mini protocols outlining how fidelity could be maintained while also accommodating contextual issues could be considered. It may, for example, mean allocating testing to a particular nurse or HCA who sees the value in offering testing, finds the test easy to deliver and enjoys new tasks, or (in busy practices) extending the time allocated to the New Patient Health Check, at least in the early stages.

Another, more general way for research teams to guard against poor uptake of interventions by participating units is to address the issue of slack resources. Perhaps unsurprisingly, successful practices in our trial tended to have greater slack resource (staff time) to incorporate rapid testing into their regular practices. The issue here is that the time taken for one staff member to do the test is only part of the total time needed to assimilate the intervention. A practice with limited slack resources has no alternative but to pull staff from research activities when deadlines are looming for other key priorities. Specific packages of back-fill or overtime pay might be key to continuing to deliver at these times of stress.

Some low-recruiting practices raised concerns about leadership, staff relations, role distributions and possible internal hostilities. Such issues make the routinisation of innovations extremely difficult, and it may be that sensitive exploration of the system antecedents and key success factors for implementation (Fig. 1) may allow practices with such ‘risky’ characteristics to be identified in advance of the trial and offered targeted support (or even excluded from the sampling frame).

The question of who makes the decision for a practice to participate in a complex intervention trial is key. As Everett Rogers observed, the adoption decision for an innovation can be optional (i.e., everyone can freely decide), collective (i.e., everyone in an organisation or team must commit) or authoritarian (i.e., imposed by more powerful members of an organisation on the less powerful) [22]. Authoritarian decisions lead to high rates of initial adoption but also to high levels of front-line resistance, particularly in practices where human resource issues are already present. Ideally, the decision for an organisation to join a trial of a complex intervention should be made collectively and should certainly include the staff whose job it will be to deliver the intervention.

### Strengths and limitations of the study

We used an evidence-based model of the diffusion of health care innovations and applied it to qualitative and quantitative data from a cluster RCT. The wide variation in HIV rapid test uptake in the trial enabled testing of the model, which had not been previously applied to a trial design. Detailed qualitative and quantitative data allowed us to examine model aspects and their interplay through four contrasting case studies. However, the model was applied retrospectively and in a single trial. We recommend further new, complex intervention trials replicating the approach as well as applying the model prospectively (perhaps with additional degrees of complexity such as behavioural interventions) to extend understanding of the model’s applicability. We have not produced a simple checklist for universal application, because any such checklist would need to be developed to accommodate both ‘hard core’ and ‘soft periphery’ elements specific to the intervention being studied.

## Conclusions

An adaptation of the diffusion of innovations framework was an effective analytical tool for retrospectively explaining high-recruiting and low-recruiting practices in a complex intervention research trial. A better understanding of aspects that may hinder or promote uptake and routinisation may support the improved delivery of interventions such as diagnostic screening in primary care settings. Whether the model will work prospectively to predict performance (and hence shape the design of future trials) is unknown.

## Abbreviations

EMIS: Egton Medical Information Systems
GP: General practitioner
HCA: Health care assistant
MRC: Medical Research Council
NIHR: National Institute for Health Research
RCT: Randomised controlled trial
